# Plasma neurofilament light chain is associated with clinical instability in chronic autoimmune neuropathies

**DOI:** 10.1038/s41598-026-39803-x

**Published:** 2026-02-16

**Authors:** Ieva Glāzere, Marija Roddate, Violeta Žukova, Nataļja Kurjāne, Kaj Blennow, Henrik Zetterberg, Viktorija Ķēniņa

**Affiliations:** 1 Department of Neurology, Pauls Stradiņš Clinical University Hospital, Riga, Latvia; 2 Department of Doctoral Studies, Rīga Stradiņš University, Riga, Latvia; 3 Department of Biology and Microbiology, Rīga Stradiņš University, Riga, Latvia; 4 Center for Clinical Immunology and Allergology, Pauls Stradiņš Clinical University Hospital, Riga, Latvia; 5 Institute of Oncology and Molecular Genetics, Rīga Stradiņš University, Riga, Latvia; 6 Department of Psychiatry and Neurochemistry, Institute of Neuroscience and Physiology, the Sahlgrenska Academy at the University of Gothenburg, Mölndal, Sweden; 7 Clinical Neurochemistry Laboratory, Sahlgrenska University Hospital, Mölndal, Sweden; 8 Department of Neurodegenerative Disease, UCL Institute of Neurology, Queen Square, London, UK; 9 UK Dementia Research Institute at UCL, London, UK; 10 Hong Kong Center for Neurodegenerative Diseases, Clear Water Bay, Hong Kong China; 11 Wisconsin Alzheimer’s Disease Research Center, University of Wisconsin School of Medicine and Public Health, University of Wisconsin-Madison, Madison, WI USA

**Keywords:** Chronic inflammatory demyelinating polyneuropathy, Multifocal motor neuropathy, Autoimmune neuropathies, Biomarkers, Neurofilament light chain, Biomarkers, Diseases, Neurology, Neuroscience

## Abstract

Neurofilament light chain (NfL) is a sensitive biomarker of axonal damage, but its clinical relevance in chronic autoimmune neuropathies remains incompletely defined. This study evaluated plasma NfL levels in patients with chronic inflammatory demyelinating polyneuropathy (CIDP) and multifocal motor neuropathy (MMN), compared with hereditary neuropathy (Charcot–Marie–Tooth disease type 1 A, CMT1A) and healthy controls, focusing on disease activity rather than diagnostic discrimination. Plasma NfL concentrations were measured using single molecule array (Simoa) technology in 41 patients (CIDP *n* = 16, MMN *n* = 7, CMT1A *n* = 18) and 25 age- and sex-matched controls. Disease severity was assessed using the Inflammatory Rasch-built Overall Disability Scale for autoimmune neuropathies and the Charcot–Marie–Tooth Neuropathy Score version 2 for CMT. Plasma NfL levels were significantly higher in patients with autoimmune neuropathies and CMT compared with controls. No significant differences were observed between inflammatory and hereditary neuropathies, and NfL levels did not correlate with disability scores. However, patients with autoimmune neuropathies and an unstable disease course, defined by more than two relapses, exhibited significantly higher plasma NfL levels. Across all groups, NfL concentrations showed a strong correlation with age. These findings suggest that while plasma NfL lacks diagnostic specificity among chronic neuropathies, it may be associated with disease instability in autoimmune neuropathies.

## Introduction

Chronic inflammatory demyelinating polyneuropathy (CIDP) and multifocal motor neuropathy (MMN) are immune-mediated disorders of the peripheral nervous system characterised by chronic or relapsing disease courses. Chronic inflammatory demyelinating polyneuropathy typically presents with progressive or relapsing motor and sensory deficits, whereas multifocal motor neuropathy is characterised by asymmetric, purely motor weakness without sensory involvement, and shows a characteristic response to immunoglobulin therapy. Despite advances in diagnostic criteria and disease classification, both conditions remain challenging to diagnose and monitor in routine clinical practice.

Epidemiological data indicate that CIDP and MMN are rare diseases, with a prevalence in Latvia of approximately 1.21 and 0.42 per 100,000 individuals, respectively. However, their clinical impact is substantial due to progressive disability, frequent relapses, and the need for long-term immunomodulatory treatment. Diagnostic uncertainty remains common, particularly in differentiating CIDP from hereditary neuropathies and MMN from motor neuron disorders. Misdiagnosis rates remain high and can result in delayed or inappropriate treatment, exposing patients to unnecessary risks and healthcare systems to substantial costs^1,2^.

The introduction of updated European Federation of Neurological Societies/Peripheral Nerve Society (EFNS/PNS) guidelines has improved the classification of CIDP and its variants through refined clinical and electrophysiological criteria^3^. Nevertheless, diagnostic accuracy still relies heavily on clinical expertise and neurophysiological testing, while objective blood-based biomarkers are lacking. Furthermore, tools to monitor disease activity, axonal damage, and long-term progression in chronic autoimmune neuropathies remain limited.

Neurofilament light chain (NfL) is a structural protein of the neuronal cytoskeleton and a well-established marker of neuroaxonal injury^4^. Elevated levels of NfL in cerebrospinal fluid and blood have been reported in a wide range of neurological conditions, including neurodegenerative, inflammatory, and traumatic disorders^5,6^. In peripheral neuropathies, increased NfL concentrations are thought to reflect axonal damage secondary to demyelination or immune-mediated injury^7,8^. Although several studies have demonstrated elevated NfL levels in both acquired and hereditary neuropathies, its clinical value for disease differentiation, activity assessment, and progression monitoring in chronic autoimmune neuropathies remains uncertain.

Therefore, the aim of this study was to evaluate plasma NfL levels in patients with CIDP and MMN in comparison with hereditary neuropathy (Charcot–Marie–Tooth disease type 1 A) and healthy controls, with particular emphasis on disease activity, relapse history, and clinical stability rather than diagnostic discrimination alone.

## Materials and methods

### Study population

This cross-sectional study included 41 patients and 25 healthy control subjects. The patient cohort comprised 16 individuals diagnosed with chronic inflammatory demyelinating polyneuropathy, 7 with multifocal motor neuropathy, and 18 with hereditary neuropathy (Charcot–Marie–Tooth disease type 1 A, CMT1A).

CIDP was diagnosed according to the 2021 European Federation of Neurological Societies/Peripheral Nerve Society criteria; MMN was diagnosed according to the revised EFNS/PNS criteria and was considered a purely motor immune-mediated neuropathy, in contrast to CIDP, which may involve both motor and sensory fibres. CMT diagnoses were confirmed by genetic testing^1,9^. Healthy controls had no history or clinical signs of neurological disease and were matched to the patient groups by age and sex.

Disease severity in patients with autoimmune neuropathies was assessed using the Inflammatory Rasch-built Overall Disability Scale (I-RODS), while severity in patients with CMT was evaluated using the Charcot–Marie–Tooth Neuropathy Score version 2 (CMTNSv2)^10^. Clinical data, including disease duration, treatment status, and relapse history, were obtained from medical records.

The study was conducted in accordance with the Helsinki Declaration of 1975, as revised in 2008 and approved by the Central Medical Ethics Committee of Latvia approval Nr.3/18-03-21. Informed consent was obtained from all subjects involved in the study.

### Blood sampling and processing

Venous blood samples were collected by certified medical personnel in an outpatient setting using ethylenediaminetetraacetic acid (EDTA) tubes. Samples were processed within 1 h of collection by centrifugation at 3500 rpm for 10 min at 20 °C. Plasma was aliquoted and stored at − 20 °C until analysis.

Plasma neurofilament light chain (NfL) concentrations were measured using a commercially available single-molecule array (Simoa) assay on the HD-X Analyzer (Quanterix, Billerica, MA, USA), following the manufacturer’s instructions and previously published protocols^11^.

### Statistical analysis

Data distribution was assessed visually and using normality tests, which indicated non-normality. Therefore, nonparametric statistical methods were applied. Group comparisons were performed using the Kruskal–Wallis H test, followed by pairwise comparisons with the Mann–Whitney U test where appropriate.

Correlations between plasma NfL concentrations and clinical variables were assessed using Spearman’s rank correlation coefficient. Given the known age dependence of plasma NfL levels, correlations with age were evaluated separately in each group.

Statistical analyses were performed using Jamovi (version 2.3), and statistical significance was set at *p* < 0.05.

## Results

### Characteristics of the patient and control groups

A total of 41 patients and 25 healthy controls were included in the study. The autoimmune neuropathy group consisted of 23 patients, including 16 diagnosed with chronic inflammatory demyelinating polyneuropathy and 7 with multifocal motor neuropathy. The hereditary neuropathy group comprised 18 patients with genetically confirmed Charcot-Marie-Tooth disease type 1 A.

In the autoimmune neuropathy group, 11 patients were female, and 12 were male, with a mean ± standard deviation (SD) age of 53.0 ± 19.4 years. The median Inflammatory Rasch-built Overall Disability Scale (I-RODS) score was 36 (interquartile range [IQR] 17). The CMT group included nine women and nine men, with a mean ± SD age of 43.1 ± 16.3 years and a median Charcot–Marie–Tooth Neuropathy Score version 2 (CMTNSv2) of 13 (IQR 10.5).

The control group consisted of 25 healthy individuals (11 women and 14 men) with a mean ± SD age of 42.9 ± 12.1 years. No statistically significant differences in age or sex distribution were observed between the patient groups and controls. Baseline clinical characteristics of patients with autoimmune neuropathies, including disease duration, relapse history, and treatment status, are summarised in Table 1.

**Table 1:** Baseline characteristics of the autoimmune neuropathy group.

ID	Neuropathy type∗	Sex (1 = female 2 = male)	Disease duration (months)	Course of the disease (1: >2 relapses; 2: <2 relapses)	Treatment	I-RODS score
1	Typical CIDP	1	40–45	2	Subcutaneous immunoglobulin	36
2	Sensory-predominant CIDP	2	45–50	1	Subcutaneous immunoglobulin	47
3	Typical CIDP	2	55–60	2	Subcutaneous immunoglobulin	36
4	Multifocal CIDP	1	35–40	1	Subcutaneous immunoglobulin	46
5	Typical CIDP	1	> 100	2	Subcutaneous immunoglobulin	43
6	Typical CIDP	1	45–50	1	Subcutaneous immunoglobulin	39
7	Typical CIDP	2	55–60	2	Subcutaneous immunoglobulin	30
8	Typical CIDP	2	55–60	1	Subcutaneous immunoglobulin	48
9	Typical CIDP	2	65–70	2	Subcutaneous immunoglobulin	9
10	Typical CIDP	1	> 100	1	Subcutaneous immunoglobulin	28
11	Typical CIDP	2	< 20	2	None	18
12	Typical CIDP	2	45–50	1	Subcutaneous immunoglobulin	38
13	Typical CIDP	2	< 20	1	None	31
14	Typical CIDP	2	35–40	2	Subcutaneous immunoglobulin	33
15	Sensory-predominant CIDP	2	35–40	1	Subcutaneous immunoglobulin	42
16	Typical CIDP	1	> 100	2	Subcutaneous immunoglobulin	24
17	Definitive MMN	1	55–60	1	Subcutaneous immunoglobulin	19
18	Definitive MMN	1	40–45	1	Subcutaneous immunoglobulin	34
19	Definitive MMN	2	70–75	1	Subcutaneous immunoglobulin	44
20	Definitive MMN	1	45–50	2	Subcutaneous immunoglobulin	43
21	Definitive MMN	2	35–40	1	Intravenous immunoglobulin	3
22	Definitive MMN	1	55–60	2	Subcutaneous immunoglobulin	22
23	Definitive MMN	1	45–50	1	Subcutaneous immunoglobulin	47

∗According to European Federation of Neurological Societies/Peripheral Nerve Society (EFNS/PNS) guidelines. CDIP, chronic inflammatory demyelinating polyneuropathy; I-RODS, Inflammatory Rasch-built Overall Disability Scale; MMN, multifocal motor neuropathy.

### Neurofilament light chain concentration in patients and the control group

**Fig. 1:**
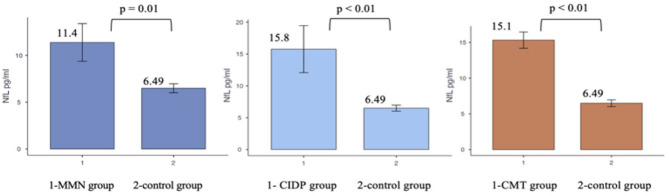
Plasma neurofilament light chain (NfL) concentrations in the autoimmune neuropathy, Charcot–Marie–Tooth (CMT), and control groups. The autoimmune neuropathy group includes patients with chronic inflammatory demyelinating polyneuropathy (CIDP) and multifocal motor neuropathy (MMN).

Plasma neurofilament light chain concentrations differed significantly between patients with neuropathies and healthy controls. Both the autoimmune neuropathy and CMT1A groups demonstrated significantly higher plasma NfL levels than healthy controls (Fig. 1).

No statistically significant differences in plasma NfL concentrations were observed between the autoimmune neuropathy group and the hereditary neuropathy group (Kruskal–Wallis test, χ² = 5.03, *p* = 0.081). Similarly, plasma NfL concentrations did not differ significantly between patients with CIDP and MMN; therefore, these groups were analysed together in subsequent analyses. Median plasma NfL concentrations for each patient group are presented in Table 2.

**Table 2:** Comparison of plasma neurofilament light chain (NfL) concentrations for the patient groups.

	CIDP group	MMN group	CMT group	Difference between the groups (Kruskal–Wallis test)
NfL level (pg/mL), median (IQR)	9.88 (6.78)	10.4 (5.12)	15.1 (4)	χ²=5.03; degrees of freedom = 2 *p* = 0.081

CIDP, chronic inflammatory demyelinating polyneuropathy; CMT, Charcot–Marie–Tooth disease; IQR, interquartile range; MMN, multifocal motor neuropathy.

### Association of plasma NfL with disability, age, and disease duration

Plasma NfL concentrations did not correlate significantly with clinical disability measures. No correlation was observed between plasma NfL levels and I-RODS scores in patients with autoimmune neuropathies (Spearman rs = 0.07, *p* = 0.78), nor between plasma NfL levels and CMTNSv2 scores in patients with CMT1A (rs = 0.09, *p* = 0.73).

In contrast, plasma NfL concentrations demonstrated a significant positive correlation with age in all study groups, including patients with autoimmune neuropathies (rs = 0.58, *p* = 0.004), patients with CMT1A (rs = 0.62, *p* = 0.006), and healthy controls (rs = 0.73, *p* < 0.01).

Plasma NfL concentrations did not correlate significantly with disease duration in patients with autoimmune neuropathies (rs = 0.08, *p* = 0.71), indicating that cumulative disease duration alone does not account for elevated NfL levels.

### Plasma NfL and disease course in autoimmune neuropathies

Within the autoimmune neuropathy group, plasma NfL concentrations were significantly higher in patients with an unstable disease course, defined as a history of more than 2 relapses, compared with those with a more stable course (*p* = 0.004). This classification was applied exclusively to autoimmune neuropathies and not to patients with CMT1A, who do not exhibit relapses or disease instability.

This association was independent of disease duration and disability scores, suggesting that elevated plasma NfL levels reflect ongoing or recurrent axonal injury rather than static neurological impairment.

## Discussion

The present study demonstrates that plasma neurofilament light chain levels are elevated in patients with chronic autoimmune neuropathies and hereditary demyelinating neuropathy, reflecting axonal injury rather than disease-specific pathology. Our findings do not support the use of NfL as a diagnostic or differential diagnostic biomarker to distinguish between chronic inflammatory demyelinating polyneuropathy, multifocal motor neuropathy, and Charcot-Marie-Tooth disease type 1 A. Instead, the results indicate that plasma NfL serves as a marker of neuroaxonal damage and may provide clinically relevant information regarding disease activity and instability in autoimmune neuropathies.

NfL is a structural protein of the neuronal cytoskeleton and plays a critical role in maintaining axonal integrity. Increased concentrations of NfL in cerebrospinal fluid and blood have been consistently reported in neurological disorders characterised by axonal damage, including multiple sclerosis, Alzheimer’s disease, and motor neuron disease^5^. In these conditions, elevated NfL levels reflect widespread neurodegeneration and ongoing axonal injury, supporting its role as a biomarker of disease activity and progression rather than disease aetiology^5,12^. Axonal injury is also a common pathological feature of peripheral neuropathies. In predominantly demyelinating disorders such as CIDP and CMT1A, the primary pathological process involves myelin loss, which disrupts saltatory conduction and ultimately leads to secondary axonal degeneration^13,14^. This secondary axonal damage is a significant determinant of long-term disability and provides a plausible biological explanation for increased plasma NfL levels observed in these conditions^4,8,11^.

A key finding of the present study is the higher plasma neurofilament light chain (NfL) concentrations observed in patients with autoimmune neuropathies who had a clinically unstable disease course, defined by more than 2 relapses. This association was not explained by disease duration or clinical disability scores, suggesting that elevated NfL levels may reflect ongoing or recurrent neuroaxonal injury rather than cumulative damage or fixed neurological impairment. These observations indicate that plasma NfL may be more informative about disease instability than cross-sectional measures of disease severity in chronic immune-mediated neuropathies. However, given the study’s cross-sectional design, causal or temporal relationships cannot be established, and the findings should therefore be interpreted as exploratory and hypothesis-generating.

Consistent with previous studies, plasma NfL levels were significantly higher in patients with CIDP, MMN, and CMT1A compared with healthy controls^7,15,16^. However, no significant differences were observed between inflammatory and hereditary neuropathy groups, nor between CIDP and MMN. These findings indicate that while NfL is sensitive to axonal injury, it lacks specificity for differentiating between distinct types of peripheral neuropathies. This is in line with the existing literature, which demonstrates that NfL performs best as a biomarker in conditions characterised by widespread or rapidly progressive neuroaxonal damage, such as amyotrophic lateral sclerosis, and in selected acute neurological conditions, such as distinguishing ischaemic stroke from transient ischaemic attack^17,18^. In contrast, chronic peripheral neuropathies share overlapping mechanisms of axonal injury, which limits the discriminatory capacity of NfL across diagnostic categories.

In the present study, plasma NfL levels did not correlate significantly with clinical disability scores, as assessed by I-RODS in autoimmune neuropathies and CMTNSv2 in CMT1A. Several studies have questioned the clinical utility of blood neurofilament light chain (NfL) in slowly progressive peripheral neuropathies, reporting limited prognostic value and uncertain cost-effectiveness in routine clinical practice. These observations suggest that, in chronic neuropathies, NfL is more likely to reflect cumulative or ongoing axonal injury rather than functional impairment or short-term disease progression, thereby limiting its ability to reliably capture clinical severity in slowly progressive or chronically treated conditions. Similar conclusions have been reported by Kodal et al., who noted the limited prognostic value of blood NfL in slowly progressive polyneuropathies and questioned its cost-effectiveness in this context^12^. This contrasts with disorders such as multiple sclerosis, in which NfL levels correlate more closely with disease activity and progression, likely reflecting the complex interplay of inflammatory demyelination, astroglial activation, and neurodegeneration that characterises central nervous system pathology^19^.

Taken together, these findings indicate that plasma neurofilament light chain (NfL) is a sensitive but non-specific marker of axonal injury in peripheral neuropathies. Although NfL does not support differential diagnosis between autoimmune and hereditary neuropathies, higher plasma NfL levels observed in patients with clinically unstable autoimmune disease suggest a potential association with ongoing neuroaxonal injury. Given the study’s cross-sectional design and the strong age dependence of plasma NfL, these observations should be interpreted cautiously and regarded as exploratory. Larger studies incorporating age-adjusted multivariable analyses, prospective longitudinal sampling, and standardised treatment timing will be required to determine whether plasma NfL can reliably contribute to monitoring disease dynamics or identifying patients at increased risk of clinical instability in chronic autoimmune neuropathies.

## Limitations

This study has several limitations that should be considered when interpreting the results. The sample size was relatively modest, particularly in the multifocal motor neuropathy subgroup, limiting disease-specific subgroup analyses and precluding multivariable regression models that adjust for potential confounders, such as age and treatment status. Nevertheless, the cohort size is comparable to that of other biomarker studies in rare peripheral neuropathies.In addition, the study cohort was characterised by a predominance of patients with typical CIDP, with a lower proportion of CIDP variants than expected based on epidemiological data. This distribution likely reflects referral patterns in a tertiary neuromuscular centre and may limit the generalisability of the findings to CIDP variants. The small size of the MMN subgroup further limited statistical power for disease-specific analyses. Moreover, multiple exploratory comparisons were performed without formal correction for multiple comparisons, thereby increasing the risk of Type I error. Accordingly, the findings should be interpreted cautiously and require confirmation in larger, independent cohorts.

Second, plasma neurofilament light chain levels are known to be influenced by age. Although age-matched controls were included and age-related correlations were analysed, formal age-adjusted multivariable analyses were not performed due to the limited sample size, particularly within disease subgroups. As a result, residual confounding by age cannot be fully excluded, and the observed association between disease instability and plasma NfL levels should be interpreted cautiously.

Third, most patients with autoimmune neuropathies were receiving immunoglobulin therapy at the time of blood sampling, and the interval between the last treatment administration and sample collection varied across individuals. Given the limited sample size and heterogeneity of treatment timing, treatment-stratified or time-adjusted analyses were not feasible. Consequently, potential short-term effects of immunoglobulin therapy on plasma NfL levels cannot be excluded. In addition, the cross-sectional design of the study precludes conclusions regarding longitudinal changes in NfL concentrations or their temporal relationship with clinical relapses and treatment response.

Finally, disease duration was heterogeneous across the patient cohort. Although no correlation between disease duration and plasma NfL levels was observed, longitudinal studies with repeated sampling are needed to clarify whether changes in NfL levels precede clinical relapses or reflect cumulative axonal damage over time.

## Data Availability

The raw data supporting the conclusions of this article will be made available by the authors upon a reasonable request.
